# The Significance of Tumor Necrosis Factor Receptor Type II in CD8^+^ Regulatory T Cells and CD8^+^ Effector T Cells

**DOI:** 10.3389/fimmu.2018.00583

**Published:** 2018-03-22

**Authors:** Lin-Lin Ye, Xiao-Shan Wei, Min Zhang, Yi-Ran Niu, Qiong Zhou

**Affiliations:** Department of Respiratory Medicine, Union Hospital, Tongji Medical College, Huazhong University of Science and Technology, Wuhan, China

**Keywords:** tumor necrosis factor, tumor necrosis factor receptor type II, CD8^+^ regulatory T cells, CD8^+^ effector T cells, CD4^+^ regulatory T cells

## Abstract

Tumor necrosis factor (TNF) is a pleiotropic cytokine that has both pro-inflammatory and anti-inflammatory functions. The biological functions of TNF are mediated by two receptors, TNF receptor type I (TNFR1) and TNF receptor type II (TNFR2). TNFR1 is expressed universally on almost all cell types and has been extensively studied, whereas TNFR2 is mainly restricted to immune cells and some tumor cells and its role is far from clarified. Studies have shown that TNFR2 mediates the stimulatory activity of TNF on CD4^+^Foxp3^+^ regulatory T cells (Tregs) and CD8^+^Foxp3^+^ Tregs, and is involved in the phenotypic stability, proliferation, activation, and suppressive activity of Tregs. TNFR2 can also be expressed on CD8^+^ effector T cells (Teffs), which delivers an activation signal and cytotoxic ability to CD8^+^ Teffs during the early immune response, as well as an apoptosis signal to terminate the immune response. TNFR2-induced abolition of TNF receptor-associated factor 2 (TRAF2) degradation may play an important role in these processes. Consequently, due to the distribution of TNFR2 and its pleiotropic effects, TNFR2 appears to be critical to keeping the balance between Tregs and Teffs, and may be an efficient therapeutic target for tumor and autoimmune diseases. In this review, we summarize the biological functions of TNFR2 expressed on CD8^+^Foxp3^+^ Tregs and CD8^+^ Teffs, and highlight how TNF uses TNFR2 to coordinate the complex events that ultimately lead to efficient CD8^+^ T cell-mediated immune responses.

## Introduction

Tumor necrosis factor (TNF) is a pleiotropic cytokine involved in regulating diverse functions, including cell growth modulation, viral replication, septic shock, tumorigenesis, inflammation, and autoimmunity (1, 2). These functions hinge upon the binding of TNF to two distinct membrane receptors on target cells: TNF receptor (TNFR) 1and TNFR2. TNFR1 is expressed universally on almost all cell types, whereas TNFR2 is restricted to immune cells (2–6) and some tumor cells (7–13). Since TNFR1 and TNFR2 were identified (14), multiple studies have been carried out to characterize their structures and functions. While TNFR1 has been extensively characterized, the biological functions of TNFR2 have remained elusive (15). There is mounting evidence to suggest that TNFR2 is expressed on and has critical roles in immune cells, including CD4^+^ regulatory T cells (Tregs) (16), CD4^+^ effector T cells (Teffs) (4), CD8^+^ Tregs (17), and CD8^+^ Teffs (18). This implies that TNFR2 is involved in various T cell-mediated immune responses. TNFR2 expressed on CD4^+^ T cells has been studied in depth with many studies indicating that TNFR2 mediates the stimulatory activity of TNF on CD4^+^ Treg cells, resulting in their phenotypic stability, proliferation, and activation (3, 19–22). Furthermore, TNFR2 can be used to identify the maximally suppressive subset of CD4^+^ Tregs (20). However, studies on TNFR2 expression on CD8^+^ T cells are relatively deficient. Several studies have identified TNFR2 as a potent costimulatory molecule on CD8^+^ T cells required to sustain cell survival and protect from apoptosis, while TNFR2 expressing CD8^+^Foxp3^+^ Tregs exhibited highly suppressive activity (17, 23, 24).

The restricted distribution of TNFR2 has identified it as a potential target for immunotherapy. Targeting TNFR2 for cancer immunotherapy has seen remarkable success. Treatment of OVVAR3, an ovarian cancer cell line with surface expression of TNFR2, with a TNFR2 antagonist induced significant tumor cell death. Furthermore, the TNFR2 antagonist preferentially suppressed the activity of tumor-associated CD4^+^ Treg cells, but had little inhibitory effects on peripheral CD4^+^ Treg cells or cells from healthy donors (25). This result indicates that patients treated with a TNFR2 antagonist can maintain immunological homeostasis and mitigate the collateral damage to healthy tissues (20, 25). While the potential effects of TNFR2 antagonists on tumors have been documented, major questions remain unanswered, including how much the effects of therapeutically targeting TNFR2 *in vivo* are directly related to modulating T cell activity. Better knowledge of the fundamental biological processes, such as signaling pathway activation and the molecular mechanism underlying the T cell response to TNFR2 stimulation, especially in Treg cells, may help design safer and more effective targeted therapeutics. As TNFR2 expression on CD4^+^ T cells has been documented in detail, in this review, we mainly summarize and discuss the biological effects of TNFR2 expression on CD8^+^Foxp3^+^ Tregs and CD8^+^ Teffs.

## TNFR2 Expressed on CD8^+^ Tregs

**Figure 1:**
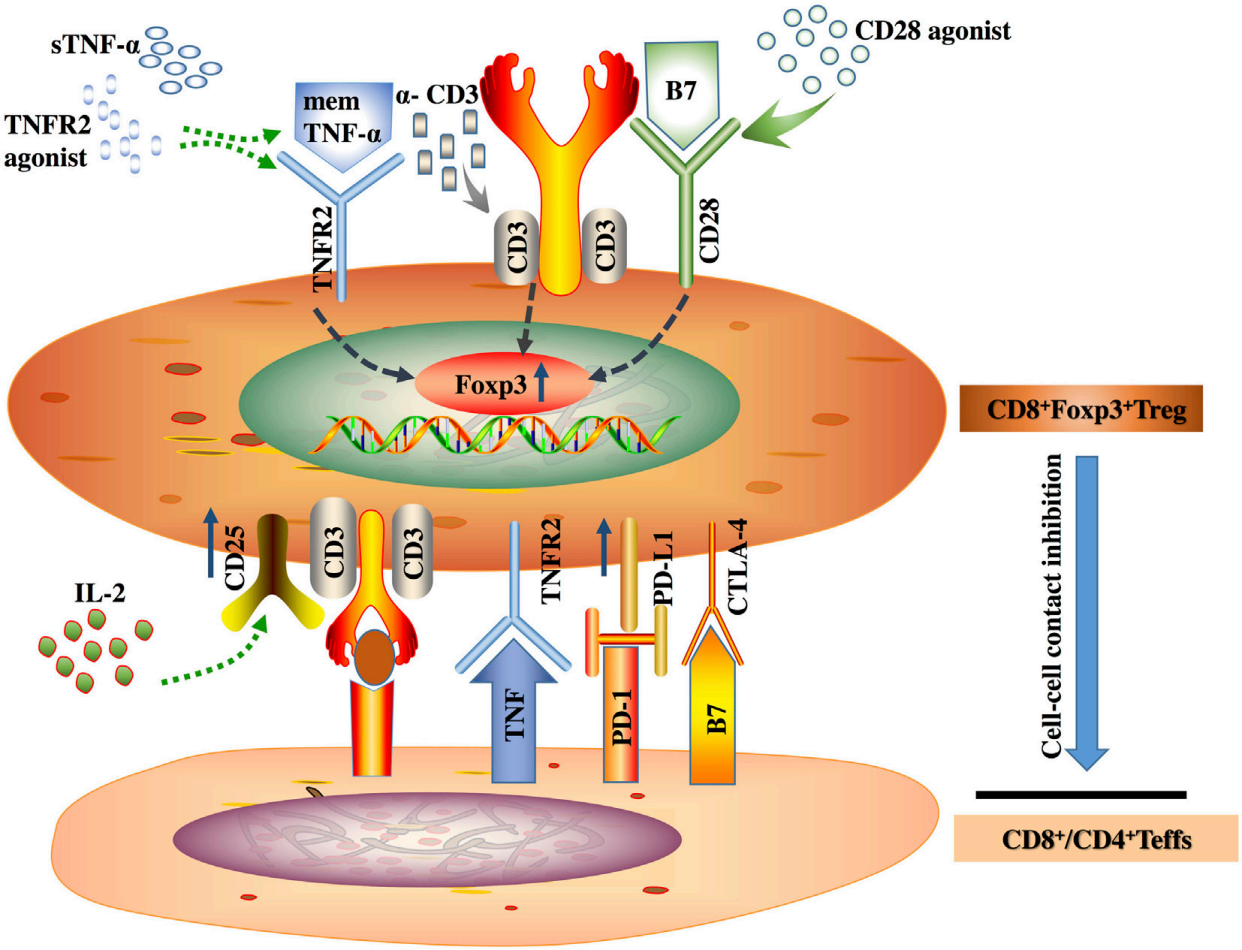
Tumor necrosis factor (TNF) receptor type II (TNFR2) acts as a suppressive marker for CD8^+^ regulatory T (Tregs) cells. The TNF/TNFR2 interaction, as well as TNFR2 and CD28 agonists, could promote the induction of Foxp3 in the presence of anti-CD3. Additionally, the TNF/TNFR2 interaction could also upregulate CD25 and PD-L1, the negative molecules on the surface of CD8^+^ Tregs, to mediate a contact-dependent inhibition to CD4^+^ and CD8^+^ effector T cells, cooperation with other negative molecules on the surface of CD8^+^ Tregs, such as CTLA-4.

The suppressive effects of CD8^+^ Tregs on normal and pathologic immune responses are well described (Figure 1) (26–28). Previous study demonstrated that human CD8^+^CD25^+^ Tregs share many features with CD4^+^CD25^+^ Tregs in the thymus, such as phenotype, function, and mechanisms of action (23). Increasing evidence suggests that TNFR2 is a significant biomarker for highly potent suppressive Tregs, because TNFR2 promotes the activation, expansion, and survival of CD4^+^ Tregs by mediating the effect of TNF (29). However, most studies on TNFR2 expression on Tregs have focused on the CD4^+^ Tregs population, rather than CD8^+^ Tregs. Current results suggest that TNFR2 might also be a critical suppressive maker of the functional CD8^+^Foxp3^+^ Tregs. However, CD8^+^ Tregs are not the CD8^+^ counterpart of CD4^+^ Tregs. There are multiple subsets of CD8^+^ Tregs reported in both humans and mice (30), such as CD8^+^CD122^+^ Tregs (31), CD8^+^CD28^−^ Tregs (32, 33), and CD8^+^CD103^+^ Tregs (34, 35). Unfortunately, the published studies on TNFR2 expression on CD8^+^Tregs all focused on CD8^+^Foxp3^+^ Tregs. As a consequence, we can only summarize the biological effects of TNFR2 expressed on CD8^+^Foxp3^+^ Tregs.

### TNFR2 Is a Better Functional Treg Cell Marker Than CD25 for CD8^+^Foxp3^+^ Tregs

CD8^+^Foxp3^+^ Tregs can be generated *in vitro* with anti-CD3 antibodies (17, 36, 37) or anti-CD3/28 beads (24). These cells expressed CD25, Foxp3, TNFR2, and the negative co-stimulatory receptors CTLA-4, PD-1, PDL-1, and Tim-3 (24). When CD8^+^ T cells were isolated from peripheral blood mononuclear cells (PBMCs) from healthy donors and cultured with anti-CD3 mAb for 5 days, the TNFR2^+^CD25^+^ cells were identified as the main subset that expressed Foxp3 (17). Similarly, human CD25 and TNFR2-coexpressing CD4^+^ Tregs were identified as a potent subpopulation of Tregs (22, 38–40). Interestingly, when these CD8^+^Tregs were sorted into four subsets, CD25^+^TNFR2^+^, CD25^+^TNFR2^−^, CD25^−^TNFR2^+^, and CD25^−^TNFR2^−^, to identify their respective ability to inhibit proliferation of target CD4^+^ Teffs, the results identified that both CD8^+^CD25^+^ and CD8^+^CD25^−^ cells were more potent inhibitors of proliferation if they coexpressed TNFR2, suggesting that TNFR2 is a more important marker than CD25 on CD8^+^Foxp3^+^ Tregs (17). Additionally, *in vitro*-induced CD8^+^Foxp3^+^ Tregs expressed both TNFR2 and PDL-1. When sorting CD8^+^ T cells into TNFR2^+^PDL-1^+^, TNFR2^+^PDL-1^−^, TNFR2^−^PDL-1^+^, or TNFR2^−^PDL-1^−^, it was observed that TNFR2-PDL-1 double positive cells exhibited much stronger suppressive activity than control sham sorted cells. TNFR2 or PDL-1 single positive cells had modest suppressive activity, while the double negative cells had none (24). Once more, these data emphasized that TNFR2 might be a characteristic expression marker for functional CD8^+^Foxp3^+^ Tregs and the coexpression of TNFR2 and PDL-1 on CD8^+^Foxp3^+^ Tregs may represent cells with stronger suppressive activity.

### TNF/TNFR2 Interaction Delivers a Co-Stimulatory Signal to Induce Foxp3 by CD8^+^Foxp3^+^ Tregs

It was shown that Foxp3 appears to function as a master regulator of the regulatory pathway in the development and function of Tregs (41–43). Interestingly, the TNF/TNFR2 interaction on the surface of CD8^+^ T cells could promote the induction of Foxp3 in the presence of anti-CD3/CD28 beads to generate more CD8^+^Foxp3^+^ Tregs. Previous studies have shown that when PBMCs from rheumatoid arthritis (RA) patients were cultured with anti-CD3 for 24 h, a greater percentage of CD8^+^Foxp3^+^ Tregs were generated and expressed high levels of CD25 and TNFR2 (44). However, when anti-TNF monoclonal antibodies (mAb) were added into the *in vitro* culture system, the percentage of Foxp3 expression on CD8^+^ Tregs decreased significantly (44). Furthermore, experimental results show that membrane TNF/TNFR2 interactions, in combination with CD80/CD28 interactions between monocytes and CD8^+^ T cells from RA patients, could also promote the induction of CD8^+^Foxp3^+^ Tregs *in vitro*, while combined CD86 and TNF blockade completely ablated the process (44). These data all indicated that the effect mediated by TNFR2 expression on CD8^+^ T cells played a prominent role for the generation of CD8^+^Foxp3^+^ Tregs in the presence of anti-CD3 *in vitro*. However, a defined mechanism remains elusive and the corresponding process *in vivo* remains to be studied.

### TNF/TNFR2 Interactions Mediate the Suppressive Activity of CD8^+^Foxp3^+^ Tregs

Tumor necrosis factor was also found to be responsible for the induction of CD8^+^Foxp3^+^ Tregs, as anti-TNF monoclonal antibodies (mAb) could dramatically abrogate the proliferation of CD8^+^Foxp3^+^ Tregs, prevent the upregulation of CD25 in response to anti-CD3 *in vitro* on CD8^+^ Tregs, and interfere with the suppressive activity of CD8^+^Foxp3^+^ Tregs. Furthermore, TNFR2 expression was upregulated significantly after CD8^+^Foxp3^+^ Tregs were stimulated with anti-CD3 mAb *in vitro*, whereas the TNFR1 level was relatively low (17), indicating that the effect of TNF was more potent *via* TNFR2 to mediate the downstream signal. Additionally, TNF could upregulate PDL-1 expression on CD8^+^Foxp3^+^ Tregs *via* TNFR2 and which was greatly decreased by blocking with soluble TNF receptors (TNFR2-Fc) (17). Therefore, upregulating PDL-1 expressing on CD8^+^ Tregs might be a specific mechanism for TNF/TNFR2 mediating CD8^+^ Treg suppressive activation (45). Compared with TNFR2 expressed on CD4^+^ Tregs, little is known about the significance of TNFR2 on CD8^+^ Tregs. The available evidence indicates that TNFR2 expression on CD8^+^Foxp3^+^ Tregs is beneficial for their function, and defects in their suppressive function occurred when TNFR2 was neutralized. CD8^+^ Tregs have been shown to exhibit different phenotypes in different diseases, including viral infection (46), autoimmune diseases (47), graft-versus-host disease (GVHD) (48, 49), and cancer (44, 50). However, it is unclear whether TNFR2 can be used as a suppressive marker for all the reported CD8^+^ Treg subsets.

## TNFR2 Expressed on CD8^+^ Effector T Cells

**Figure 2:**
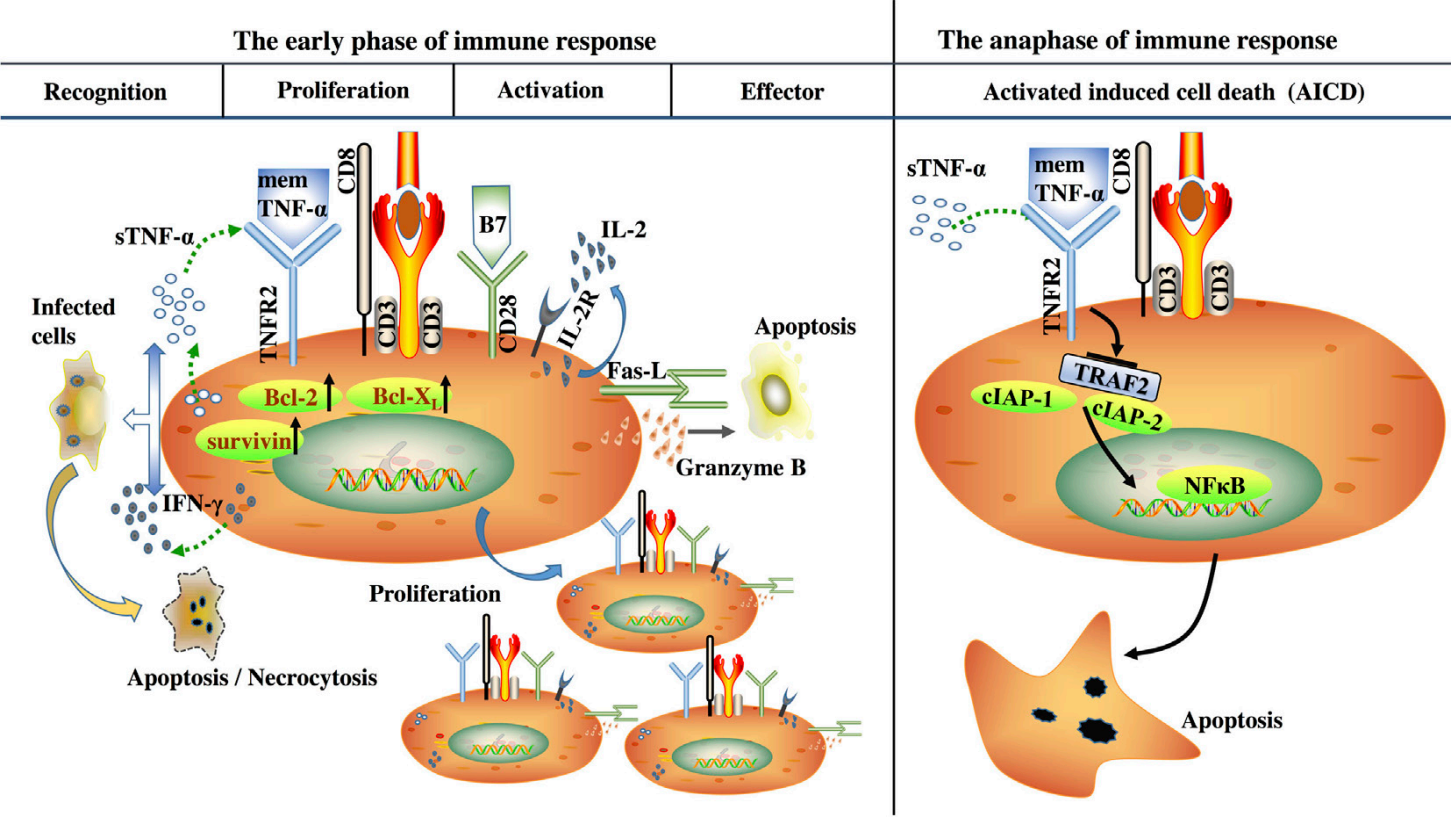
Tumor necrosis factor (TNF) receptor type II (TNFR2) modulates the process of immune response mediated by CD8^+^ effector T cells (Teffs). In the early phase of immune response, TNFR2 is a CD8^+^ Teff costimulatory molecule for IL-2, survivin, Bcl-2, and Bcl-x_L_ induction to promote CD8^+^ Teffs survival, proliferation, and activation and involve in controlling the cell fate during TCR/CD28-mediated stimulation. TNFR2 is essential for the production of IFN-γ and TNF-α in CD8^+^ Teffs to promote antigen clearance. Additionally, TNFR2 is also required for CD8^+^ Teffs to upregulate Fas-L and granzyme B to enhance their cytotoxic activity. However, in the anaphase of immune response, TNFR2 can induce activated CD8^+^ T cells programmed cell death to terminate the immune response *via* the degradation of a pro-survival signal—TRAF2, which is required for the recruitment of cellular inhibitor of apoptosis proteins cIAP-1 and cIAP-2 to the TNFR2 signaling complex and activates nuclear factor-κB.

Studies on TNFR2 expressed on CD8^+^ Teffs are relatively more sufficient than studies on TNFR2 expressed on CD8^+^ Tregs (Figure 2). Numerous reports have shown that CD8^+^ Teffs are critical players involved in various immune responses (51–53). Efficient induction of CD8^+^ Teffs requires coordinated signaling through a number of pathways, including T cell receptor (TCR) ligation with peptide in the context of major histocompatibility complex class I (MHC I), costimulatory molecules, and cytokines(53). TNFR2, but not TNFR1, has been previously shown to be the predominant TNF receptor on activation CD8^+^ Teffs (54, 55). Thus, the direct effects of TNF on CD8^+^ Teffs are mainly mediated through TNFR2 (54, 55). Typically, T-cell-mediated immune responses can be divided into three parts: (1) antigen recognition; (2) proliferation and differentiation; and (3) activation-induced cell death (AICD). Once an activation signal has been received, primary CD8^+^ T cells undergo proliferation, expansion, and differentiation. It has been reported that TNFR2 expression was involved in CD8^+^ Teffs activation in certain phases of an immune response. For instance, it has been found that TNFR2 not only lowered the threshold for T cell activation, but also provided early costimulatory signals during T cell activation (56–58). Additionally, TNFR2 also plays a critical role in regulating AICD in activated CD8^+^ Teffs (59).

### TNFR2 as an Activator Molecule in the Early Phase of Immune Response

#### TNFR2 Is Required for Primary CD8^+^ T Cell Survival and Proliferation

CD28 is a key costimulatory molecule for IL-2 induction, based on its ability to substantially augment expression in T cells stimulated *via* the TCR (60). However, the effects mediated by CD28 were found to be insufficient to sustain long-term T cell survival (61). Nevertheless, TNFR2 plays a critical role in promoting activation and survival of naive T cells during a primary response (5, 62). CD8^+^ T cells deficient in TNFR2 possessed a marked defect in IL-2 production, a critical T cell growth factor (63, 64), resulting in a decreased proliferative response (57), suggesting that TNFR2 is a CD8^+^ T cell costimulatory molecule involved in controlling the cell fate during TCR/CD28-mediated stimulation (57). Additionally, TNFR2 deficiency in CD8^+^ T cells increased the requirements for a TCR agonist, approximately fivefold to achieve a proliferative response equivalent to wild-type CD8^+^ T cells in several infection models (5, 56–58). Additionally, in a mouse tumor model, the proportion of proliferating transgenic tumor-specific CD8^+^ T cells in TNFR2 deficient mice were significant reduced in tumor-draining lymph nodes (54). These data indicated that TNFR2 sustained the early proliferative phase during CD8^+^ T cell cells activation. Moreover, during CD8^+^ T cell activation in response to antigen *in vitro*, TNFR2 deficiency was related to a reduction of anti-apoptotic molecules, such as survivin, Bcl-2, and Bcl-x_L_ (57, 58), indicating the critical roles of TNFR2 in CD8^+^ T cell survival.

#### TNFR2 Is Required for the Secretion of Effector Molecules by CD8^+^ Teffs

One of the key effector functions of activated CD8^+^ T cells is the ability to produce antiviral and pro-inflammatory cytokines, including interferon (IFN)-γ and TNF-α (65). Typically, cytokine production by antiviral CD8^+^ T cells occurs in a hierarchical fashion, with the majority producing IFN-γ, and a subset of those producing TNF-α (66–68). During infection, such as respiratory influenza or *C. muridarum* infection, the production of IFN-γ was significantly decreased in TNFR2^−/−^CD8^+^ T cell, with significantly delayed antigen clearance in TNFR2^−/−^ mice (69, 70). These results suggest that TNFR2 primarily promotes the activation of CD8^+^ T cells and enhances the ability of CD8^+^ T cells to clear antigen. When tumor-special CD8^+^ T cells, isolated from TNFR2^−/−^ mice, TNFR1^−/−^, or wild-type mice, were cultured with specific antigens *in vitro*, IFN-γ levels produced by TNFR2^−/−^CD8^+^ T cells was less than TNFR1^−/−^ or wild-type CD8^+^ T cells (54), indicating that TNFR2 was also necessary for the optimal production of IFN-γ to clear tumor antigens during the T cell activation phase.

Second, TNF-α is increased during CD8^+^ T cell activation following antigenic stimulation (71, 72). Similar to IFN-γ, TNF-α is critically required for efficient CD8^+^ T cell-mediated responses from initiation to pathogen clearance. However, TNF-α levels produced by CD8^+^ T cells were not always in line with INF-γ production. During colitis, CD8^+^ T cells from TNFR2^−/−^ mice expressed significantly higher levels of TNF-α compared with wild-type mice, which was sufficient to worsen colonic inflammation (73). Similarly, after intranasal challenge with HKx31 influenza A virus, TNF-α production was also increased in TNFR2^−/−^ mice, compared with wild-type mice (73). It is possible that the increased TNF-α in TNFR2 deficient mice may be due to a negative feedback loop in the TNF-TNFR2 signaling (5, 59, 62, 73).

#### TNFR2 Is Required for CD8^+^ T Memory Cells Recovery

After encountering with microbial antigen, T cells can differentiate into memory cells to provide long-lasting protection against subsequent pathogens (18, 74, 75). During transplantation, microbe-elicited T memory cells can also cross-react with allogeneic antigen and mediate graft rejection, a process termed allogeneic heterologous immunity. TCR affinity is hypothesized to be critically important in the context of allogeneic heterologous immunity (18, 76, 77). Notably, TNFR2 plays an important role for low-affinity-primed memory CD8^+^ T cells mediating optimum recall responses. During heterologous rechallenge, low-affinity-primed memory effectors upregulated TNFR2 surface expression to mediated graft rejection, whereas blockade of TNFR2 significantly attenuated graft rejection and prolonged graft survival (18). These data indicated that TNFR2 is required and critical for memory CD8^+^ T cells recovery in immune responses.

#### TNFR2 Is Required for Cytotoxic T Lymphocyte (CTL) Activity

Granzyme B is a serine protease expressed by CTL and together with the pore forming protein, perforin, mediates apoptosis in target cells (78). Notably, TNFR2 engagement with TNF-α induces the expression of granzyme B in CD8^+^ T cells, when costimulation with CD86 is provided simultaneously. TNFR2 was also shown to be upregulated on granzyme B^+^CD8^+^ T cells in aging mice and humans (79), indicating that the TNF/TNFR2 signaling pathway in CD8^+^ T cell could reinforce the cells’ cytotoxic activity to induce target cells apoptosis *via* the release of granzyme B.

A second way for CTL to induce apoptosis is *via* cell-surface Fas–Fas ligand (FasL) interactions between CTL and infected cells. FasL is expressed predominantly on activated lymphocytes and is able to induce programmed cell death in most Fas-expressing cells (80, 81). The number of FasL-expressing CD8^+^ intrahepatic lymphocytes isolated from various strains of hepatic adenovirus-infected TNFR2^−/−^ mice were found to be significantly reduced compared with wild-type mice (82). Furthermore, TNFR2^−/−^ intrahepatic lymphocytes were significantly less efficient in killing adenovirus-infected hepatocyte target cells than intrahepatic lymphocytes obtained from adenovirus-infected wild-type mice (82). These data provide evidence suggesting that TNFR2 can potentiate FasL-mediated cytotoxicity for CD8^+^ Teffs.

### TNFR2 Is as an Apoptosis Signal on Activated CD8^+^ Teffs

Tumor necrosis factor receptor type II is essential for both optimal proliferation during CD8^+^ T cell activation and for the induction of AICD that terminates the proliferative response (59). Previous study had shown that TNFR2^−/−^CD8^+^ T cells exhibited consistently high resistance to AICD, leading to worsen colonic inflammation (73), indicating that TNFR2 is a critical negative regulator of activated CD8^+^ T cells by promoting AICD to terminate the immune response. Moreover, TNFR2 signaling was reported to lead to the degradation of TNF receptor-associated factor 2 (TRAF2) (83), which were important in the regulation of the receptor signaling (83–86). Notably, TRAF2 is known as a pro-survival signal (87), which is required for the recruitment of cellular inhibitor of apoptosis proteins (cIAP)-1 and -2 to the TNFR2 signaling complex (88) and activates nuclear factor (NF)-κB to mediate its anti-apoptotic effects (89–91). The overexpression of TRAF2 in wild-type CD8^+^ T cells did not affect the percentage of apoptotic cells, whereas the silencing of TRAF2 in activated TNFR2^−/−^CD8^+^ T cells could render them as sensitive to AICD as activated wild-type CD8^+^ T cells (59). Collectively, these results provide evidence that the TNFR2 signaling pathway is involved in regulating AICD and that TRAF2 depletion induced by TNFR2 is critical to this process.

## Conclusion

Tumor necrosis factor receptor type II is an attractive molecular marker to identify both CD8^+^Foxp3^+^ Tregs and CD8^+^ Teffs. For CD8^+^Foxp3^+^ Tregs, TNFR2 is necessary for the induction of Foxp3 and regarded as a functional marker of their suppressive ability. For CD8^+^ Teffs, TNFR2 serves as an activator for proliferation and cytotoxic ability in the early stage of an immune response and as an apoptosis signal for activated CD8^+^ Teffs to terminate the immune response. Both CD8^+^Foxp3^+^ Tregs and CD8^+^ Teffs could express high levels of TNFR2 and were involved in various diseases. It is noteworthy that there is an antagonistic relationship between CD8^+^ Tregs and CD8^+^ Teffs. The TNF-TNFR2 signaling pathway potentially activates both of them, so targeting TNFR2 may impair the function of protective Tregs or Teffs as a side effect in the treatment of diseases (4). Furthermore, studies have shown that TNFR2 is a potential therapeutic target with remarkable success in cancer immunotherapy. A TNFR2 antagonists could specifically inhibit CD4^+^Foxp3^+^ Tregs expansion in the tumor microenvironment, whereas it had little inhibitory effects on CD4^+^ Tregs in periphery or from healthy donors, and killed human ovarian tumor cells directly. However, little is known about TNFR2 agonists or antagonists aimed at altering TNFR2 expression on tumor-associated CD8^+^ Tregs and CD8^+^ Teffs. Further understanding of TNFR2 expression on CD8^+^ T cells and the pathways that are active and important in different disease-related microenvironments will provide better understanding of its impacts on TNF-mediated pathology, and may help in the development of more effective targeted therapeutics.

Furthermore, recent evidence indicated that the relationship between TNF/TNFR2 and T cell responses is complex and, at times, paradoxical. There is controversy to the specific effects of TNF on different T cell subsets (92). The explanation for such contradictory outcomes may lay in how downstream signaling pathways are activated and drive disease (92). Consequently, a precise understanding of the level and/or ratio of TNFR2 expressed on different T cell subsets will help in the use of TNFR2 agonists or antagonists as therapies.

## Author Contributions

L-LY and QZ contributed to the design and writing of this review. X-SW, MZ, and Y-RN contributed to collection of references.

## Conflict of Interest Statement

The authors declare that the research was conducted in the absence of any commercial or financial relationships that could be construed as a potential conflict of interest.
